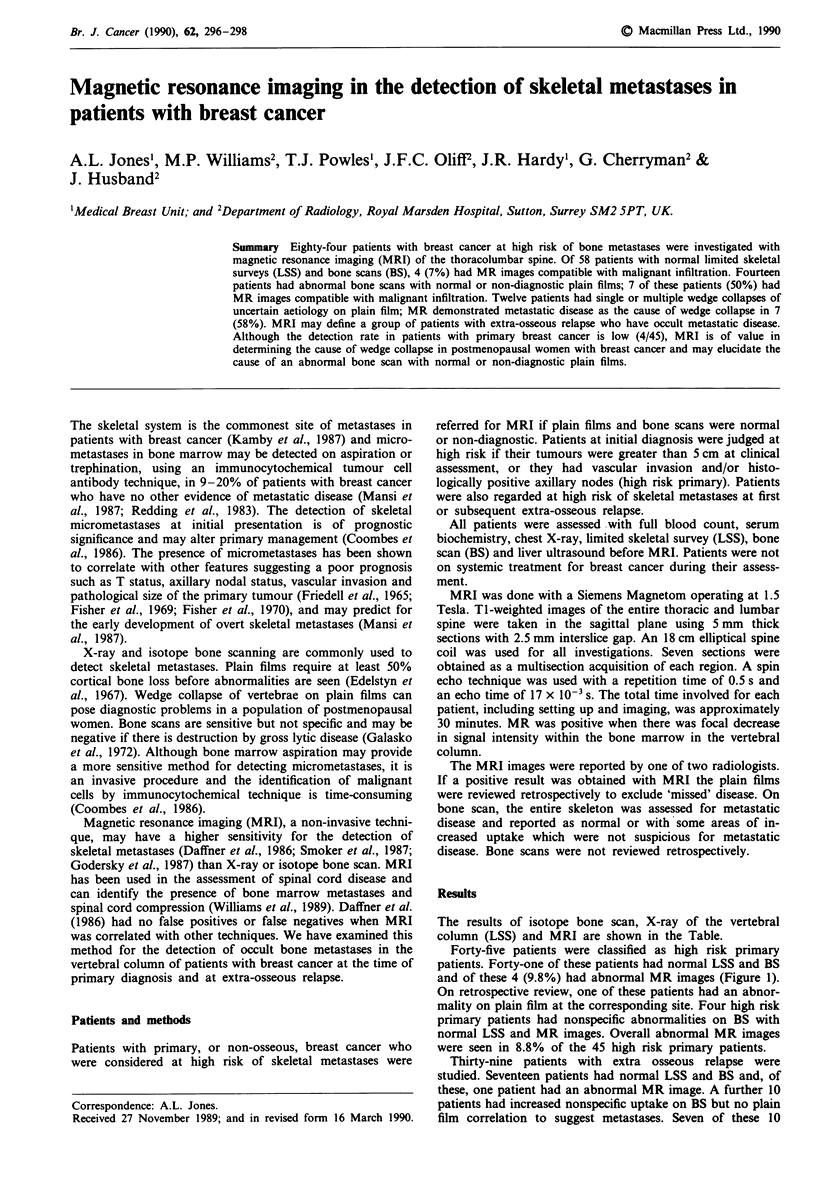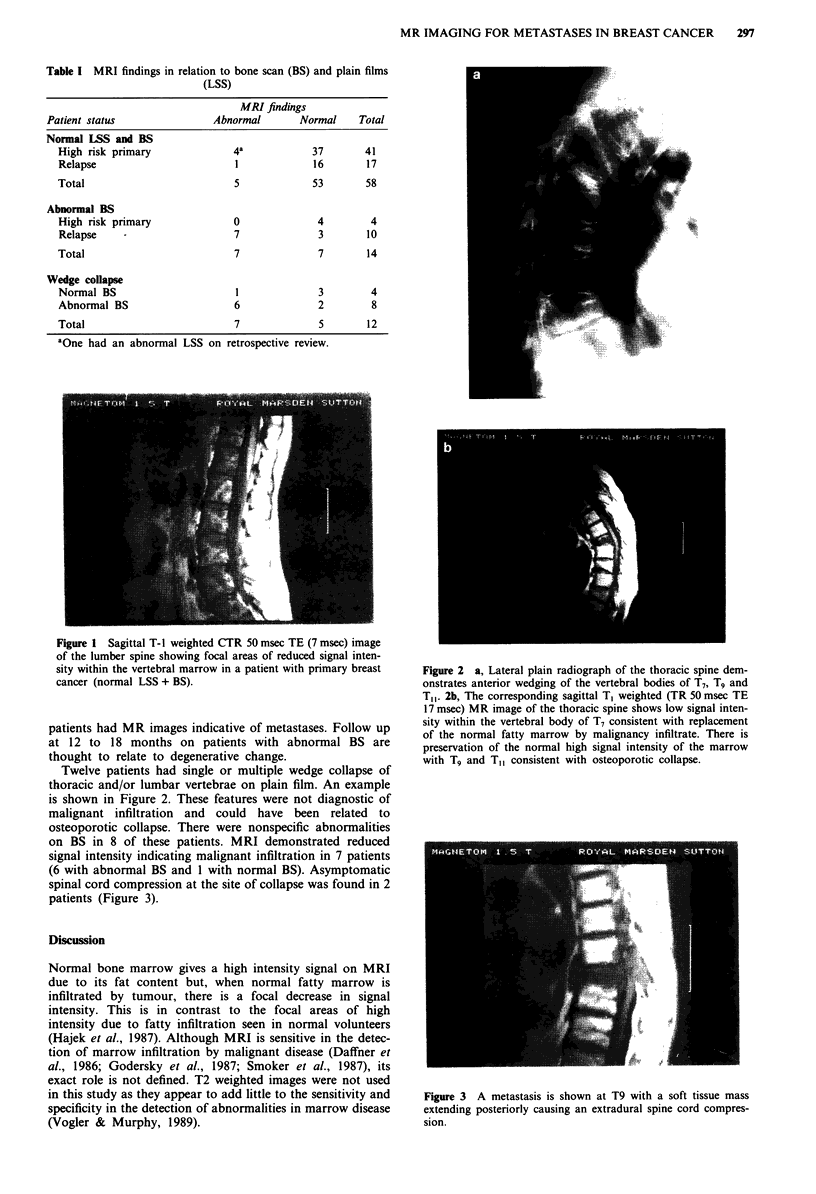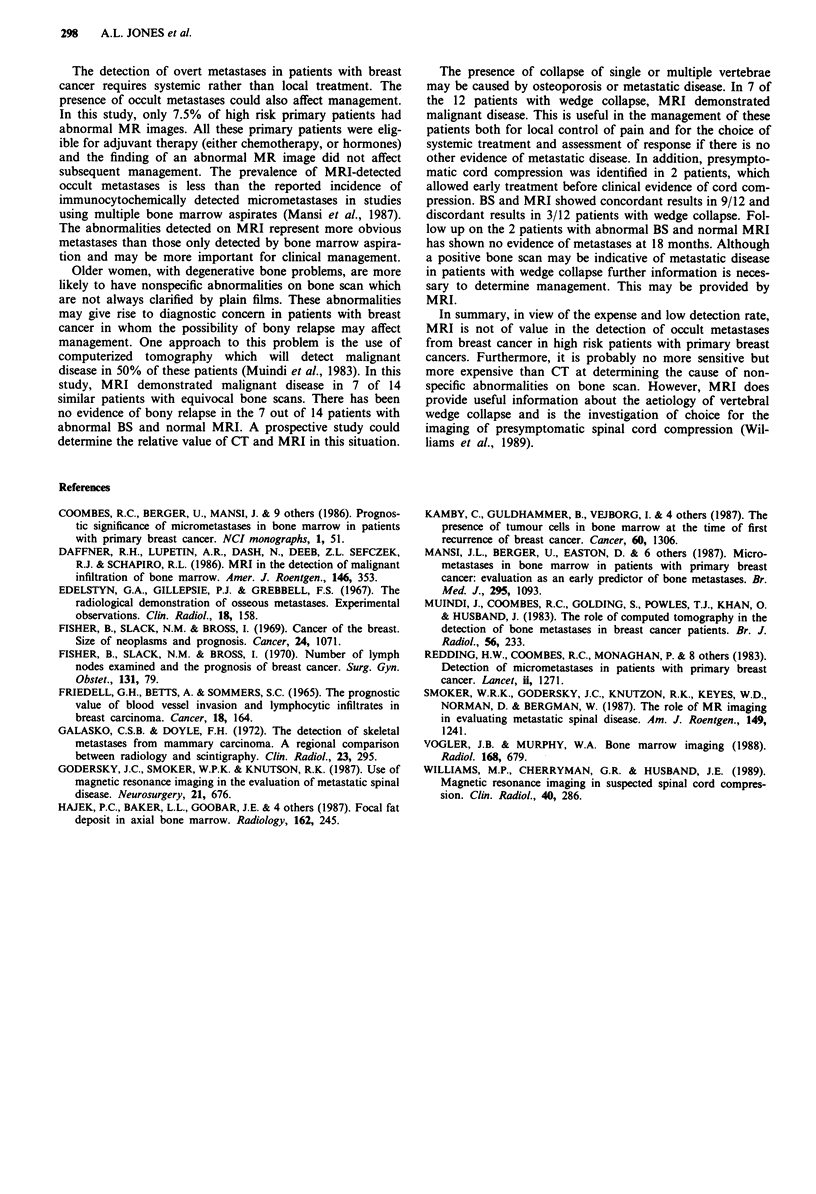# Magnetic resonance imaging in the detection of skeletal metastases in patients with breast cancer.

**DOI:** 10.1038/bjc.1990.281

**Published:** 1990-08

**Authors:** A. L. Jones, M. P. Williams, T. J. Powles, J. F. Oliff, J. R. Hardy, G. Cherryman, J. Husband

**Affiliations:** Medical Breast Unit, Royal Marsden Hospital, Sutton, Surrey, UK.

## Abstract

**Images:**


 © Macmillan Press Ltd., 1990

### Magnetic resonance imaging in the detection of skeletal metastases in patients with breast cancer

A.L. Jones[1], M.P. Williams[2], T.J. Powles[1], J.F.C. Oliff[2], J.R. Hardy[1], G. Cherryman[2] & J. Husband[2]

[1]*Medical Breast Unit; and* [2]*Department of Radiology, Royal Marsden Hospital, Sutton, Surrey SM2 5PT, UK.*

**Summary** Eighty-four patients with breast cancer at high risk of bone metastases were investigated with magnetic resonance imaging (MRI) of the thoracolumbar spine. Of 58 patients with normal limited skeletal surveys (LSS) and bone scans (BS), 4 (7%) had MR images compatible with malignant infiltration. Fourteen patients had abnormal bone scans with normal or non-diagnostic plain films; 7 of these patients (50%) had MR images compatible with malignant infiltration. Twelve patients had single or multiple wedge collapses of uncertain aetiology on plain film; MR demonstrated metastatic disease as the cause of wedge collapse in 7 (58%). MRI may define a group of patients with extra-osseous relapse who have occult metastatic disease. Although the detection rate in patients with primary breast cancer is low (4/45), MRI is of value in determining the cause of wedge collapse in postmenopausal women with breast cancer and may elucidate the cause of an abnormal bone scan with normal or non-diagnostic plain films.

The skeletal system is the commonest site of metastases in patients with breast cancer (Kamby *et al.*, 1987) and micrometastases in bone marrow may be detected on aspiration or trephination, using an immunocytochemical tumour cell antibody technique, in 9–20% of patients with breast cancer who have no other evidence of metastatic disease (Mansi *et al.*, 1987; Redding *et al.*, 1983). The detection of skeletal micrometastases at initial presentation is of prognostic significance and may alter primary management (Coombes *et al.*, 1986). The presence of micrometastases has been shown to correlate with other features suggesting a poor prognosis such as T status, axillary nodal status, vascular invasion and pathological size of the primary tumour (Friedell *et al.*, 1965; Fisher *et al.*, 1969; Fisher *et al.*, 1970), and may predict for the early development of overt skeletal metastases (Mansi *et al.*, 1987).

X-ray and isotope bone scanning are commonly used to detect skeletal metastases. Plain films require at least 50% cortical bone loss before abnormalities are seen (Edelstyn *et al.*, 1967). Wedge collapse of vertebrae on plain films can pose diagnostic problems in a population of postmenopausal women. Bone scans are sensitive but not specific and may be negative if there is destruction by gross lytic disease (Galasko *et al.*, 1972). Although bone marrow aspiration may provide a more sensitive method for detecting micrometastases, it is an invasive procedure and the identification of malignant cells by immunocytochemical technique is time-consuming (Coombes *et al.*, 1986).

Magnetic resonance imaging (MRI), a non-invasive technique, may have a higher sensitivity for the detection of skeletal metastases (Daffner *et al.*, 1986; Smoker *et al.*, 1987; Godersky *et al.*, 1987) than X-ray or isotope bone scan. MRI has been used in the assessment of spinal cord disease and can identify the presence of bone marrow metastases and spinal cord compression (Williams *et al.*, 1989). Daffner *et al.* (1986) had no false positives or false negatives when MRI was correlated with other techniques. We have examined this method for the detection of occult bone metastases in the vertebral column of patients with breast cancer at the time of primary diagnosis and at extra-osseous relapse.

### Patients and methods

Patients with primary, or non-osseous, breast cancer who were considered at high risk of skeletal metastases were referred for MRI if plain films and bone scans were normal or non-diagnostic. Patients at initial diagnosis were judged at high risk if their tumours were greater than 5 cm at clinical assessment, or they had vascular invasion and/or histologically positive axillary nodes (high risk primary). Patients were also regarded at high risk of skeletal metastases at first or subsequent extra-osseous relapse.

All patients were assessed with full blood count, serum biochemistry, chest X-ray, limited skeletal survey (LSS), bone scan (BS) and liver ultrasound before MRI. Patients were not on systemic treatment for breast cancer during their assessment.

MRI was done with a Siemens Magnetom operating at 1.5 Tesla. T1-weighted images of the entire thoracic and lumbar spine were taken in the sagittal plane using 5 mm thick sections with 2.5 mm interslice gap. An 18 cm elliptical spine coil was used for all investigations. Seven sections were obtained as a multisection acquisition of each region. A spin echo technique was used with a repetition time of 0.5 s and an echo time of $17 \times 10^{-3}$ s. The total time involved for each patient, including setting up and imaging, was approximately 30 minutes. MR was positive when there was focal decrease in signal intensity within the bone marrow in the vertebral column.

The MRI images were reported by one of two radiologists. If a positive result was obtained with MRI the plain films were reviewed retrospectively to exclude 'missed' disease. On bone scan, the entire skeleton was assessed for metastatic disease and reported as normal or with some areas of increased uptake which were not suspicious for metastatic disease. Bone scans were not reviewed retrospectively.

### Results

The results of isotope bone scan, X-ray of the vertebral column (LSS) and MRI are shown in the Table.

Forty-five patients were classified as high risk primary patients. Forty-one of these patients had normal LSS and BS and of these 4 (9.8%) had abnormal MR images (Figure 1). On retrospective review, one of these patients had an abnormality on plain film at the corresponding site. Four high risk primary patients had nonspecific abnormalities on BS with normal LSS and MR images. Overall abnormal MR images were seen in 8.8% of the 45 high risk primary patients.

Thirty-nine patients with extra osseous relapse were studied. Seventeen patients had normal LSS and BS and, of these, one patient had an abnormal MR image. A further 10 patients had increased nonspecific uptake on BS but no plain film correlation to suggest metastases. Seven of these 10

Correspondence: A.L. Jones.
Received 27 November 1989; and in revised form 16 March 1990.

**Table I** MRI findings in relation to bone scan (BS) and plain films (LSS)

| | *MRI findings* | | |
|---|---|---|---|
| *Patient status* | *Abnormal* | *Normal* | *Total* |
| **Normal LSS and BS** | | | |
| High risk primary | 4[a] | 37 | 41 |
| Relapse | 1 | 16 | 17 |
| Total | 5 | 53 | 58 |
| **Abnormal BS** | | | |
| High risk primary | 0 | 4 | 4 |
| Relapse | 7 | 3 | 10 |
| Total | 7 | 7 | 14 |
| **Wedge collapse** | | | |
| Normal BS | 1 | 3 | 4 |
| Abnormal BS | 6 | 2 | 8 |
| Total | 7 | 5 | 12 |

[a]One had an abnormal LSS on retrospective review.

**Figure 1** Sagittal T-1 weighted CTR 50 msec TE (7 msec) image of the lumber spine showing focal areas of reduced signal intensity within the vertebral marrow in a patient with primary breast cancer (normal LSS + BS).

patients had MR images indicative of metastases. Follow up at 12 to 18 months on patients with abnormal BS are thought to relate to degenerative change.

Twelve patients had single or multiple wedge collapse of thoracic and/or lumbar vertebrae on plain film. An example is shown in Figure 2. These features were not diagnostic of malignant infiltration and could have been related to osteoporotic collapse. There were nonspecific abnormalities on BS in 8 of these patients. MRI demonstrated reduced signal intensity indicating malignant infiltration in 7 patients (6 with abnormal BS and 1 with normal BS). Asymptomatic spinal cord compression at the site of collapse was found in 2 patients (Figure 3).

### Discussion

Normal bone marrow gives a high intensity signal on MRI due to its fat content but, when normal fatty marrow is infiltrated by tumour, there is a focal decrease in signal intensity. This is in contrast to the focal areas of high intensity due to fatty infiltration seen in normal volunteers (Hajek *et al.*, 1987). Although MRI is sensitive in the detection of marrow infiltration by malignant disease (Daffner *et al.*, 1986; Godersky *et al.*, 1987; Smoker *et al.*, 1987), its exact role is not defined. T2 weighted images were not used in this study as they appear to add little to the sensitivity and specificity in the detection of abnormalities in marrow disease (Vogler & Murphy, 1989).

**Figure 2** **a**, Lateral plain radiograph of the thoracic spine demonstrates anterior wedging of the vertebral bodies of $T_7$, $T_9$ and $T_{11}$. **2b**, The corresponding sagittal $T_1$ weighted (TR 50 msec TE 17 msec) MR image of the thoracic spine shows low signal intensity within the vertebral body of $T_7$ consistent with replacement of the normal fatty marrow by malignancy infiltrate. There is preservation of the normal high signal intensity of the marrow with $T_9$ and $T_{11}$ consistent with osteoporotic collapse.

**Figure 3** A metastasis is shown at T9 with a soft tissue mass extending posteriorly causing an extradural spine cord compression.

The detection of overt metastases in patients with breast cancer requires systemic rather than local treatment. The presence of occult metastases could also affect management. In this study, only 7.5% of high risk primary patients had abnormal MR images. All these primary patients were eligible for adjuvant therapy (either chemotherapy, or hormones) and the finding of an abnormal MR image did not affect subsequent management. The prevalence of MRI-detected occult metastases is less than the reported incidence of immunocytochemically detected micrometastases in studies using multiple bone marrow aspirates (Mansi et al., 1987). The abnormalities detected on MRI represent more obvious metastases than those only detected by bone marrow aspiration and may be more important for clinical management.

Older women, with degenerative bone problems, are more likely to have nonspecific abnormalities on bone scan which are not always clarified by plain films. These abnormalities may give rise to diagnostic concern in patients with breast cancer in whom the possibility of bony relapse may affect management. One approach to this problem is the use of computerized tomography which will detect malignant disease in 50% of these patients (Muindi et al., 1983). In this study, MRI demonstrated malignant disease in 7 of 14 similar patients with equivocal bone scans. There has been no evidence of bony relapse in the 7 out of 14 patients with abnormal BS and normal MRI. A prospective study could determine the relative value of CT and MRI in this situation.

The presence of collapse of single or multiple vertebrae may be caused by osteoporosis or metastatic disease. In 7 of the 12 patients with wedge collapse, MRI demonstrated malignant disease. This is useful in the management of these patients both for local control of pain and for the choice of systemic treatment and assessment of response if there is no other evidence of metastatic disease. In addition, presymptomatic cord compression was identified in 2 patients, which allowed early treatment before clinical evidence of cord compression. BS and MRI showed concordant results in 9/12 and discordant results in 3/12 patients with wedge collapse. Follow up on the 2 patients with abnormal BS and normal MRI has shown no evidence of metastases at 18 months. Although a positive bone scan may be indicative of metastatic disease in patients with wedge collapse further information is necessary to determine management. This may be provided by MRI.

In summary, in view of the expense and low detection rate, MRI is not of value in the detection of occult metastases from breast cancer in high risk patients with primary breast cancers. Furthermore, it is probably no more sensitive but more expensive than CT at determining the cause of non-specific abnormalities on bone scan. However, MRI does provide useful information about the aetiology of vertebral wedge collapse and is the investigation of choice for the imaging of presymptomatic spinal cord compression (Williams et al., 1989).

### References

COOMBES, R.C., BERGER, U., MANSI, J. & 9 others (1986). Prognostic significance of micrometastases in bone marrow in patients with primary breast cancer. *NCI monographs*, **1**, 51.

DAFFNER, R.H., LUPETIN, A.R., DASH, N., DEEB, Z.L. SEFCZEK, R.J. & SCHAPIRO, R.L. (1986). MRI in the detection of malignant infiltration of bone marrow. *Amer. J. Roentgen.*, **146**, 353.

EDELSTYN, G.A., GILLEPSIE, P.J. & GREBBELL, F.S. (1967). The radiological demonstration of osseous metastases. Experimental observations. *Clin. Radiol.*, **18**, 158.

FISHER, B., SLACK, N.M. & BROSS, I. (1969). Cancer of the breast. Size of neoplasms and prognosis. *Cancer*, **24**, 1071.

FISHER, B., SLACK, N.M. & BROSS, I. (1970). Number of lymph nodes examined and the prognosis of breast cancer. *Surg. Gyn. Obstet.*, **131**, 79.

FRIEDELL, G.H., BETTS, A. & SOMMERS, S.C. (1965). The prognostic value of blood vessel invasion and lymphocytic infiltrates in breast carcinoma. *Cancer*, **18**, 164.

GALASKO, C.S.B. & DOYLE, F.H. (1972). The detection of skeletal metastases from mammary carcinoma. A regional comparison between radiology and scintigraphy. *Clin. Radiol.*, **23**, 295.

GODERSKY, J.C., SMOKER, W.P.K. & KNUTSON, R.K. (1987). Use of magnetic resonance imaging in the evaluation of metastatic spinal disease. *Neurosurgery*, **21**, 676.

HAJEK, P.C., BAKER, L.L., GOOBAR, J.E. & 4 others (1987). Focal fat deposit in axial bone marrow. *Radiology*, **162**, 245.

KAMBY, C., GULDHAMMER, B., VEJBORG, I. & 4 others (1987). The presence of tumour cells in bone marrow at the time of first recurrence of breast cancer. *Cancer*, **60**, 1306.

MANSI, J.L., BERGER, U., EASTON, D. & 6 others (1987). Micrometastases in bone marrow in patients with primary breast cancer: evaluation as an early predictor of bone metastases. *Br. Med. J.*, **295**, 1093.

MUINDI, J., COOMBES, R.C., GOLDING, S., POWLES, T.J., KHAN, O. & HUSBAND, J. (1983). The role of computed tomography in the detection of bone metastases in breast cancer patients. *Br. J. Radiol.*, **56**, 233.

REDDING, H.W., COOMBES, R.C., MONAGHAN, P. & 8 others (1983). Detection of micrometastases in patients with primary breast cancer. *Lancet*, **ii**, 1271.

SMOKER, W.R.K., GODERSKY, J.C., KNUTZON, R.K., KEYES, W.D., NORMAN, D. & BERGMAN, W. (1987). The role of MR imaging in evaluating metastatic spinal disease. *Am. J. Roentgen.*, **149**, 1241.

VOGLER, J.B. & MURPHY, W.A. Bone marrow imaging (1988). *Radiol.* **168**, 679.

WILLIAMS, M.P., CHERRYMAN, G.R. & HUSBAND, J.E. (1989). Magnetic resonance imaging in suspected spinal cord compression. *Clin. Radiol.*, **40**, 286.